# Antibiotic Use in Livestock Farming: A Driver of Multidrug Resistance?

**DOI:** 10.3390/microorganisms13040779

**Published:** 2025-03-28

**Authors:** Andreas Matheou, Ayah Abousetta, Aphrodite Persephone Pascoe, Demosthenis Papakostopoulos, Loukas Charalambous, Stelios Panagi, Stavros Panagiotou, Andreas Yiallouris, Charalampos Filippou, Elizabeth O. Johnson

**Affiliations:** 1 School of Medicine, European University Cyprus, Nicosia 2404, Cyprus; 2 School of Medicine, Aristotle University of Thessaloniki, 54124 Thessaloniki, Greece; 3 Division of Medical Education, School of Medical Sciences, Faculty of Biology, Medicine and Health, University of Manchester, Manchester M13 9PL, UK

**Keywords:** antimicrobial resistance, multidrug resistance in livestock, One Health framework, antibiotic misuse in farming, zoonotic transmission of AMR bacteria, zoonotic pathogens, antibiotic resistance mechanisms

## Abstract

Antimicrobial resistance (AMR) constitutes a pressing and intensifying global health crisis, significantly exacerbated by the inappropriate utilization and excessive application of antibiotics in livestock agriculture. The excessive use of antibiotics, including prophylactic and metaphylactic administration as well as growth-promotion applications, exacerbates selective pressures, fostering the proliferation of multidrug-resistant (MDR) bacterial strains. Pathogens such as *Escherichia coli*, *Salmonella* spp., and *Staphylococcus aureus* can be transmitted to humans through direct contact, contaminated food, and environmental pathways, establishing a clear link between livestock farming and human AMR outbreaks. These challenges are particularly pronounced in regions with limited veterinary oversight and weak regulatory frameworks. Addressing these issues requires the implementation of sustainable practices, enhanced antibiotic stewardship, and strengthened interdisciplinary collaboration. This review underscores the critical need for a One Health approach to mitigate AMR, recognizing the interconnectedness of human, animal, and environmental health in safeguarding global public health.

## 1. Introduction

Antibiotics are indispensable in modern cattle farming, playing a crucial role in ensuring animal health, enhancing productivity, and maintaining viability of livestock operations. The use of antibiotics spans therapeutic, metaphylactic, prophylactic, and growth-promoting purposes, each addressing specific needs in livestock management.

Historically, the use of antibiotics as growth promoters significantly revolutionized livestock farming by boosting productivity and enhancing feed efficiency and disease resistance. However, growing concerns over their role in fostering antimicrobial resistance (AMR) have led to regulatory restrictions in regions like the European Union (EU) and the United States (US). Ionophores, which are not used in human medicine, remain approved for improving feed efficiency [1].

**Figure 1:**
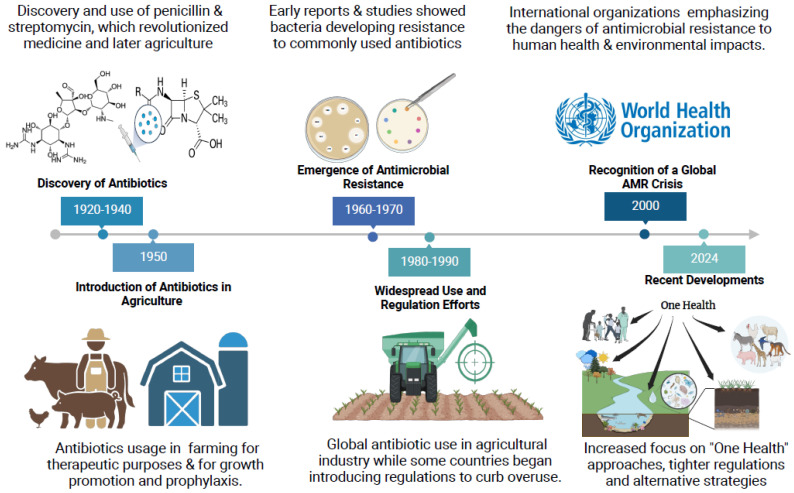
A timeline of antibiotics and antimicrobial resistance: from discovery to global challenges (created with BioRender.com, accessed on 5 January 2025) [6].

**Figure 2:**
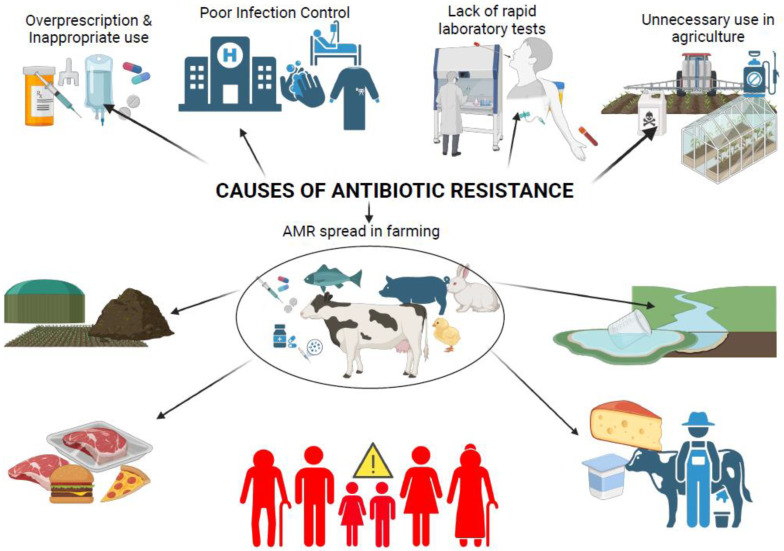
Antibiotic resistance causes and spread in Farming (created with BioRender.com, accessed on 5 January 2025).

Despite such efforts, the agricultural sector continues to face surveillance over the role of non-therapeutic antibiotic use in exacerbating AMR (Figure 1). AMR represents one of the most pressing public health challenges of the 21st century. Resistant microorganisms compromise the efficacy of existing antibiotics, jeopardizing the treatment of bacterial infections in both humans and animals. In livestock farming, excessive antibiotic use fosters the emergence of multidrug-resistant (MDR) bacteria through mechanisms such as selective pressure and environmental dissemination (Figure 2). For instance, antibiotic residues in manure, often used as fertilizer, contribute to the spread of resistance genes in soil and water systems, creating reservoirs of MDR bacteria that can affect humans and animals [2]. Additionally, recent surveillance has identified emerging pathogens, including multidrug-resistant *Klebsiella pneumoniae*, coagulase-negative staphylococci (CoNS), and *Acinetobacter baumannii* in livestock farming environments. These pathogens have been recovered from various livestock species and farming equipment, with growing evidence supporting their potential for zoonotic transmission [3,4]. Even though they are emerging in animal farming, these pathogens are well-known human pathogens responsible for severe nosocomial infections, particularly in immunocompromised patients and intensive care unit settings. The spread of these multidrug-resistant organisms from livestock to humans could exacerbate the already critical issue of antimicrobial resistance, complicating treatment options and increasing healthcare burdens. Zoonotic transmission of these pathogens will only make the problem worse, facilitating the exchange of resistance genes between human and animal reservoirs and heightening the risk of untreatable infections [5].

### 1.1. The Emergence of Multidrug Resistance (MDR) in Cattle

Antibiotics are indispensable in both human and animal health, serving as critical tools for the prevention and treatment of bacterial infections. The overlap in the use of antibiotic classes, such as β-lactams, fluoroquinolones, aminoglycosides, macrolides, and tetracyclines, highlights their shared importance across these domains (Table 1, [7]). Many of these antibiotics are designated as critically important antimicrobials (CIAs) by the WHO and as veterinary critically important antimicrobials (VCIAs) by the OIE, reflecting their essential role in managing life-threatening infections in humans and animals [8,9]. However, their widespread and often unregulated use in animals—particularly for growth promotion and disease prevention—contributes significantly to the development of cross-resistance. For instance, genes such as bla_SHV-1, ermC, and tetA/B confer resistance to antibiotics used in both sectors, emphasizing the interconnectedness of resistance mechanisms across species [7,10,11].

Multidrug resistance among pathogenic bacteria has become an escalating global health challenge, driven by their remarkable ability to adapt through intrinsic and acquired resistance mechanisms. Intrinsic resistance arises from genetic mutations, enabling bacteria to modify drug targets, neutralize antibiotics through enzymes like β-lactamases, or reduce drug uptake via altered membrane proteins. Such mechanisms are particularly notable in Gram-negative bacteria like *E. coli*, which possess protective outer membranes [12]. Acquired resistance, on the other hand, is primarily facilitated by horizontal gene transfer (HGT), involving the transfer of resistance genes through plasmids, integrons, and transposons. HGT processes, including conjugation, transformation, and transduction, allow bacteria to rapidly disseminate resistance traits, significantly accelerating MDR development [13,14]. The selective pressures due to the overuse and misuse of antibiotics further exacerbate these processes, favoring the survival and proliferation of resistant strains, particularly in agricultural systems where antimicrobial stewardship is often lacking [15]. Likewise, bacteria’s coexistence with natural antimicrobials for centuries has also driven their evolutionary capacity to resist eradication [16].

Cattle serve as a significant reservoir of MDR pathogens, including *E. coli, Salmonella* spp., and *S. aureus*. For example, studies on Nigerian cattle have reported alarming rates of extended-spectrum β-lactamase (ESBL)-producing *E. coli*, with 99% of isolates exhibiting MDR [17]. Similarly, methicillin-resistant *S. aureus* (MRSA) has been identified in dairy cattle in regions such as China and Ethiopia, further highlighting the global threat of MDR [18,19]. The spread of MDR pathogens is exacerbated by factors such as poor farming practices, inadequate sanitation, and the interaction between exposed and unexposed cattle [20]. Regional differences in MDR prevalence reflect variations in antimicrobial use, veterinary oversight, and farming systems [21].

### 1.2. Selective Pressures Driving MDR

Globally, livestock accounts for approximately 73% of total antimicrobial use (AMU), with the majority of this usage occurring in low- and middle-income countries (LMICs) due to inadequate regulatory frameworks and weak veterinary systems [8,21]. Antibiotics such as beta-lactams, tetracyclines, and aminoglycosides are heavily utilized in cattle production, significantly contributing to the development of resistance. For instance, a study in Kenya revealed that over 95% of veterinary antibiotics were obtained over-the-counter and administered without professional oversight, primarily for therapeutic purposes. This lack of supervision is compounded by the absence of enforced national policies, facilitating unchecked opportunities for resistance to emerge [18].

Subtherapeutic antibiotic dosing, which is common in intensive farming systems, further exacerbates selective pressures, facilitating the development and spread of MDR pathogens [21,22]. In addition to direct selective pressures from antibiotic misuse, poor sanitation, unsanitary animal housing, and inadequate biosecurity measures in farming environments play pivotal roles in enabling the persistence and dissemination of MDR bacteria. For example, mobile genetic elements such as plasmids, integrons, and transposons carry resistance genes that spread rapidly under the influence of selective pressures created by inappropriate antimicrobial use [15]. In Ethiopia, indiscriminate antibiotic use was strongly linked to resistance in *S. aureus* isolates, with high levels of penicillin resistance (>90%) and moderate resistance to tetracycline (35.4%), reflecting the long-term impacts of selective pressure [18].

Selective pressures from antibiotic misuse in livestock are particularly conducive to the emergence of pathogens like MDR *K. pneumoniae*, coagulase-negative *staphylococci* (CoNS), and *A. baumannii*. These pathogens persist in farm environments due to inadequate sanitation, fostering reservoirs for zoonotic spread [10]. Recent studies have documented MDR *K. pneumoniae* harboring carbapenemase genes (bla_NDM, bla_OXA-48) in bovine respiratory infections, multidrug-resistant CoNS carrying mecA in cases of bovine mastitis, and *A. baumannii* isolates from raw milk and farm environments [4,23], highlighting the critical need to consider their roles in zoonotic transmission [24,25].

Weak regulatory frameworks further exacerbate the issue in LMICs, where antibiotics are frequently used without veterinary oversight or diagnostic confirmation of infections. This is particularly concerning given projections of a 50% increase in antimicrobial use in livestock by 2030. Such trends underscore the urgent need for stricter regulations and enhanced stewardship programs to mitigate misuse and limit the development of resistance [8,21].

### 1.3. Global Prevalence and Dissemination of MDR Bacteria in Cattle

The prevalence of MDR bacteria in cattle has been extensively documented, demonstrating the impact of AMR in both intensive and subsistence farming systems. In rural Nepal, 47.7% of bacterial isolates from livestock were found to be ESBL producers, with 39.4% exhibiting multidrug resistance. These findings are particularly concerning as they were observed in subsistence farms with limited antibiotic use, emphasizing the role of environmental factors and close human–livestock interactions in resistance development [22]. Similarly, a study conducted in Mekelle, Ethiopia, found that 6.2% of *S. aureus* isolates from dairy cattle exhibited MDR, with penicillin resistance exceeding 90% among these isolates. In Kenya, MDR *Staphylococcus* spp. were widespread in livestock environments, with resistance levels particularly high against beta-lactams and macrolides. The study emphasized that close contact with animals and practices such as raw milk consumption were significant risk factors for zoonotic transmission [18].

The global prevalence of MDR bacteria in cattle is driven by both direct and indirect antibiotic applications. In rural China, antibiotic-resistant bacteria and resistance genes were detected in agricultural soils treated with manure from antibiotic-fed livestock. Researchers found significant enrichment of genes conferring resistance to tetracyclines, sulfonamides, and β-lactams in these soils compared to untreated areas, suggesting the role of manure as a reservoir for AMR dissemination [26]. The study also identified mobile genetic elements, such as integrons, that facilitate HGT between bacterial populations in the environment. In the US, a multistate outbreak of MDR *Salmonella Heidelberg* (SH) linked to dairy calves illustrated the zoonotic potential of resistant bacteria. This outbreak involved strains resistant to up to eleven antibiotic classes, including ceftriaxone and ciprofloxacin, highlighting the role of animal transport and shared equipment in pathogen dissemination [20]. In Kenya, MDR *Staphylococcus* spp. were widespread in livestock environments, with resistance levels particularly high against beta-lactams and macrolides. The study emphasized that close contact with animals and practices such as raw milk consumption were significant risk factors for zoonotic transmission [18].

Beyond *E. coli*, *Salmonella* spp., and *S. aureus*, additional MDR pathogens such *as K. pneumoniae*, *A. baumannii*, and CoNS have been increasingly reported in cattle. Carbapenem-resistant *K. pneumoniae* strains harboring bla_NDM and bla_OXA-48 genes have been isolated from bovine respiratory infections, raising concerns about cross-species transmission (Table 1) [27]. Likewise, *A. baumannii* has been identified in raw milk samples from Kenya, with genomic analyses showing phylogenetic relatedness to human clinical isolates [27]. Moreover, recent surveillance data underscore global dissemination of MDR *K. pneumoniae*, notably in cattle farms across Africa and Asia [28,29]; MDR CoNS widely prevalent in European pig and dairy farms [30]; and *A. baumannii* found in raw milk in African countries [31]. The detection of these pathogens across diverse farming systems confirms their emerging zoonotic potential and further underscores the need for targeted surveillance and antimicrobial stewardship strategies to curb their emergence [19].

### 1.4. Factors Driving the Persistence and Spread of MDR Bacteria in Cattle

In many cases, antibiotic-resistant pathogens become deeply entrenched within farming systems due to inadequate biosecurity measures, unsanitary practices, and the routine prophylactic and metaphylactic use of antibiotics. The frequent use of antibiotics in young cattle, which are particularly susceptible to colonization by MDR pathogens due to their immature gut microbiota, further contributes to resistance development. The movement and mixing of cattle across farms and markets also amplify the dissemination of resistant strains, as exemplified by the *SH* outbreak in the US [20]. The persistence of MDR bacteria in livestock not only poses direct risks to animal health but also carries significant public health implications. Resistant pathogens can be transmitted to humans through multiple routes, including direct contact, consumption of contaminated meat or dairy products, and environmental exposure to soil and water polluted by animal feces. For example, Shiga toxigenic *E. coli* (STEC) strains recovered from dairy calves, animal handlers, and their surrounding environments in a study exhibited high levels of MDR and demonstrated genetic relatedness, underscoring the potential for zoonotic transmission [32]. Similarly, the recovery of ESBL-producing *Enterobacteriaceae* in both livestock and farmers in rural settings highlights the interconnected nature of resistance across humans, animals, and the environment [22].

The implications of MDR bacteria extend to both animal and human health. Commonly identified MDR pathogens in cattle include *E. coli, Salmonella* spp., *S. aureus*, *K. pneumoniae*, and *A. baumannii* [33]. In Nigeria, it was reported that 99% of *E. coli* isolates from beef cattle were ESBL producers, exhibiting resistance to multiple antibiotic classes [17]. Similar findings have been reported globally, including MRSA in dairy cattle from China and Ethiopia [18,19]. Despite the global threat posed by MDR bacteria, their prevalence exhibits regional variability, often correlated with differences in regulatory frameworks, farming practices, and resource availability. Regions with limited regulatory oversight and intensive farming systems experience higher resistance rates [21]. Addressing these disparities requires tailored antimicrobial stewardship programs that consider regional challenges and limitations. Additionally, subtherapeutic antibiotic use with limited supervision increases selective pressures, promoting intrinsic resistance and HGT of resistance genes [13,16]. Environmental factors such as poor sanitation, inadequate facility management, and the interaction between exposed and unexposed cattle further accelerate the spread of MDR pathogens [12,14]. Furthermore, the formation of biofilms by these pathogens enhances their ability to withstand antimicrobials and host immune responses, while also facilitating the exchange of antibiotic resistance genes between them [34]. These conditions establish cattle farms as significant reservoirs of MDR bacteria, facilitating the circulation of resistant pathogens and the emergence of new MDR strains.

### 1.5. Antibiotic Use in Regulated and Unregulated Farming Systems

Different countries have adopted diverse approaches to regulating antibiotic use in livestock farming. These approaches range from strict regulatory frameworks to minimal or completely absent regulations, all of which play a significant role in the development of AMR. In high-income countries, including those in the EU and the US, antibiotic use in livestock is highly regulated. In 2006, the EU banned the use of antibiotics for growth promotion and introduced strict regulations for metaphylactic antibiotic use in livestock [2]. Furthermore, the European Surveillance of Veterinary Antimicrobial Consumption (ESVAC) monitors veterinary antibiotic use to ensure alignment with international guidelines [21]. Similarly, Denmark has successfully reduced antibiotic consumption in livestock by incorporating farmer education programs and promoting alternative disease prevention strategies [35].

In the US, the Veterinary Feed Directive (VFD) mandates documentation for the use of medically important antibiotics in animal feed, as well as encouraging vaccination programs and the use of probiotics to promote growth. These efforts have significantly reduced antibiotic use and improved biosecurity [21,35]. In contrast, many low- and middle-income countries, including parts of Asia and Africa, lack adequate regulatory frameworks regarding antibiotic use in livestock. In the absence of effective regulations, antibiotics are often overused and misused, significantly increasing the risk of AMR emergence and spread [27]. Recent studies in Kenya revealed that approximately 95% of veterinary antibiotics are used without professional oversight [36]. Similarly, in Ethiopia, *Staphylococcus aureus* exhibited 90% resistance to penicillin due to extensive antibiotic use [18].

In Nigeria, *E. coli* strains producing ESBLs have been reported to show extremely high rates of MDR, reaching up to 99% [17]. Besides limited regulation, other factors also contribute to the persistence of AMR, including poor biosecurity, lack of proper sanitation, and limited awareness among farmers, all of which further drive the development of AMR. The differences in regulatory frameworks between countries directly impact AMR prevalence. Strict guidelines and effective monitoring systems contribute to controlling antibiotic use and mitigating AMR, whereas regions with weak surveillance and monitoring continue to experience increasing prevalence of MDR pathogens, posing serious public health threats [37]. The World Health Organization (WHO) has identified AMR as one of the top ten global threats to humanity, emphasizing that a collaborative global effort is needed to address these imbalances. Strengthening veterinary services, enforcing strict guidelines and policies, and promoting education and awareness are essential to achieving sustainable AMR mitigation [9,38].

## 2. Methodology

### 2.1. Literature Search

**Figure 3:**
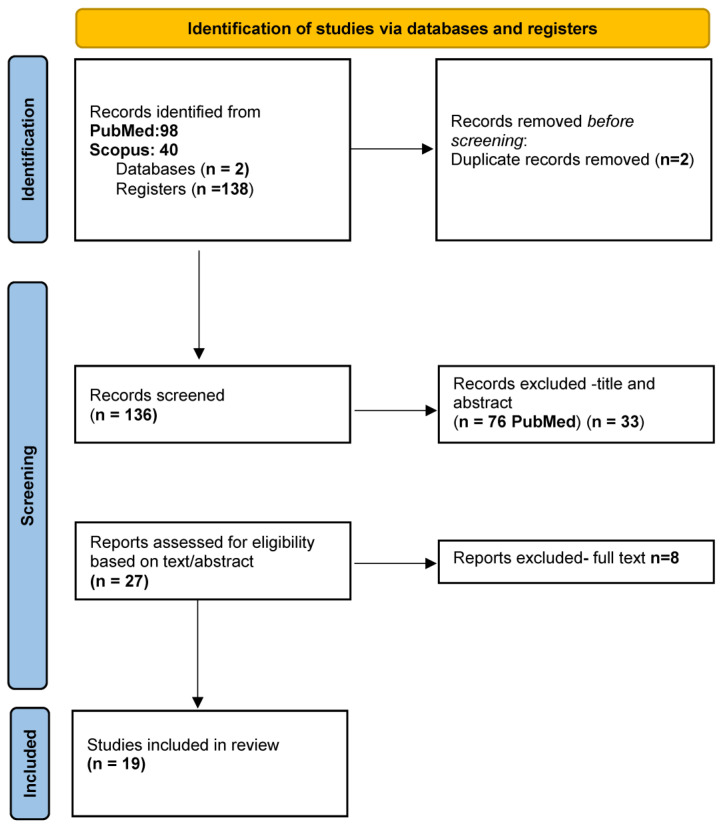
PRISMA flowchart.

For the purpose of this review, both PubMed and Scopus were selected to ensure comprehensive coverage of the relevant literature. A combination of MeSH terms and Boolean operators were used to capture relevant studies following PRISMA guidelines (Figure 3).

### 2.2. Inclusion/Exclusion Criteria

The papers selected based on specific inclusion criteria:

- Population: Studies that focus on cows or cattle receiving antibiotic treatments and studies that involve farmers or farm workers who interact with livestock.
- Intervention: Research investigating antibiotic use in cattle, particularly excessive, prolonged, or regular antibiotic administration, and studies comparing different antibiotic usage practices (e.g., organic vs. conventional, high dose vs. limited use, etc.).
- Outcome: Studies reporting the emergence of multidrug-resistant bacteria in cattle or the environment and studies examining the transmission (direct/indirect) of antibiotic-resistant bacteria from cattle to humans (e.g., farmers, farm workers, or consumers).
- Study Design: Experimental, observational, or cross-sectional studies, as well as systematic reviews, meta-analyses, cohort studies, or case–control studies.

Exclusion Criteria:

- Studies that focus solely on other livestock (e.g., pigs, poultry) without mentioning cows or cattle, as well as studies that do not involve farmers or farm environments. Additionally, studies that do not specifically focus on antibiotic use in cattle or are not related to the use of antibiotics or even focus only on resistance mechanisms unrelated to cattle or farming environment have also been excluded. Finally, studies that do not address the emergence of multidrug resistance or bacterial transmission to humans or that do not focus on bacterial resistance (e.g., they focus on viral or fungal pathogens) have also been excluded.

### 2.3. Bacterial Transmission and Resistance Mitigation Framework

#### 2.3.1. Transmission of MDR Bacteria from Cows to Humans

The discovery of ESBL-producing *E. coli* isolates in humans, cattle, and abattoir environments highlights that the transfer of resistance genes is facilitated by close contact and certain slaughtering practices. Alarmingly, resistance to last-resort antibiotics, such as colistin, has also been detected, posing serious challenges to public health and treatment outcomes [17]. Moreover, indirect exposure routes from contaminated environments [8] or the consumption of products such as raw milk [27] and undercooked meat [8] further compound these risks.

Resistant bacteria, once colonized, may lead to symptomatic infections or facilitate the transfer of resistance genes to commensal flora, thereby complicating clinical management [39]. Gut colonization of such bacteria in farmers and livestock creates a reservoir for HGT and potential outbreaks, with the high prevalence of CTX-M-15-type ESBL genes across humans, animals, and the environment underscoring the bidirectional flow of resistance in rural ecosystems [22]. The presence of MRSA ST398 on dairy farms further exacerbates public health risks, particularly due to its resistance to beta-lactam antibiotics, and contamination of raw milk and environmental factors contribute to its spread, raising concerns about food safety and zoonotic infections [40].

The zoonotic transmission potential is evident in STEC strains, which are genetically related among humans, dairy calves, and their shared environment; these MDR strains and specific sequence types have been linked to severe human infections such as hemorrhagic colitis and hemolytic uremic syndrome (HUS), highlighting significant public health threats [32]. The economic consequences are similarly severe: zoonotic outbreaks, exemplified by avian influenza, result in billions of dollars in losses and disrupt global trade [21], while projections suggest that AMR could drive a 3% to 8% reduction in livestock production annually by 2050 [8]. Despite these risks, many farmers acknowledge the importance of reducing antimicrobial use but do not perceive AMR as an immediate threat. This emphasizes the need for targeted awareness campaigns and veterinary guidance [41]. Both farmers and veterinarians often lack a full understanding of AMR’s broader implications for human health, necessitating enhanced educational initiatives [35].

The transmission of MDR bacteria from cattle to humans has significant clinical and public health implications. Colonization with resistant strains can lead to persistent asymptomatic carriage, increasing the risk of future infections that are more difficult to treat due to limited therapeutic options. Infections caused by MDR *E. coli*, *Salmonella* spp., and MRSA ST398 have been associated with prolonged hospital stays, increased morbidity and mortality, and higher healthcare costs. Moreover, genomic studies have demonstrated phylogenetic links between MDR *K. pneumoniae*, CoNS, and *A. baumannii* isolates from cattle and clinical human infections, reinforcing the zoonotic relevance of these pathogens [42]. Consequently, the presence of MDR zoonotic pathogens in the food chain also increases the likelihood of community outbreaks, disproportionately affecting vulnerable populations such as immunocompromised individuals, the elderly, and young children.

#### 2.3.2. Strategies to Mitigate Antibiotic Resistance in Cattle

Mitigating antibiotic resistance requires a holistic framework that integrates improved surveillance, informed policy interventions, and responsible antibiotic use across the livestock sector. Building upon a One Health framework that recognizes the interconnectedness of human, animal, and environmental health provides robust strategies to curb the spread of MDR pathogens. Enhanced surveillance programs are vital for monitoring resistance trends and identifying emerging threats. These require closer veterinary involvement, as well as a One Health perspective. Incorporating molecular investigations will enhance our understanding of resistance mechanisms [27]. Emphasizing the importance of antimicrobial stewardship and a One Health approach, nationwide surveillance programs and stricter regulations on antibiotic use in dairy farming are recommended to control the spread of resistant strains [18]. Central to these efforts is leveraging advanced molecular tools such as whole-genome sequencing (WGS) to track resistance genes and improve epidemiological investigations, coupled with robust surveillance systems, stricter antibiotic use regulations, and improved biosecurity measures [15]. Incorporating metagenomics and advanced phylogenetic analyses can further unravel transmission dynamics, informing targeted regulatory policies and stewardship programs aimed at reducing resistance prevalence [39].

Policy interventions such as Denmark’s reduction in antimicrobial growth promoters, supported by comprehensive monitoring, demonstrate successful strategies [21]. Enforcing regulations on antimicrobial use, banning antibiotics for growth promotion, and improving biosecurity are key measures, with evidence from high-income countries showing monitoring systems that effectively curb AMU. Strengthening veterinary services with the adoption of preventive measures like vaccination, improved husbandry, and farmer education are necessary to ensure sustainable AMU reductions [8], while education campaigns targeting livestock handlers, veterinarians, and producers can help avert future resistance-related incidents [20]. Improving farm-level biosecurity, minimizing direct human–animal interactions, and enhancing hygiene practices in dairy production systems are recommended to limit cross-species bacterial transmission [19].

Veterinarians play a pivotal role in promoting preventive measure by educating farmers about prudent AMU and AMR, with tailored training and awareness efforts particularly beneficial for younger or less experienced farmers [41]. Promoting selective dry cow therapy (SDCT), enhancing veterinary-farmer collaboration, and implementing advanced technological solutions for herd management are also identified as critical strategies [33]. Further recommendations include generating evidence-based research tailored to local contexts, utilizing rapid diagnostic tools, balancing AMU restrictions with animal welfare considerations, and enforcing stricter legislation to limit broad-spectrum antimicrobials while enhancing education for both veterinarians and farmers [43]. Finally, integrating bacteriological testing and susceptibility assessments into treatment protocols ensures a more targeted and effective use of antimicrobials [44].

Existing regulatory frameworks in HICs, such as Denmark, serve as a model for effectively addressing AMR. These frameworks emphasize comprehensive monitoring, enforced policies, and farmer education. LMICs could adapt these strategies by accounting for their unique economic and infrastructural constraints, prioritizing capacity building, and fostering international collaborations to ensure effective implementation. Mitigating antibiotic resistance in cattle farming requires a coordinated, multi-level approach rooted in the One Health framework. Effective strategies should integrate enhanced surveillance, stricter antibiotic stewardship, improved veterinary oversight, and farm-level interventions to minimize unnecessary antibiotic use while safeguarding animal health. Surveillance programs should include routine sampling from cattle, farm environments, and animal products to monitor the emergence of resistant pathogens and resistance genes. 

In addition to surveillance, strengthening regulations to restrict the non-therapeutic use of antibiotics, including growth promotion and routine prophylaxis, is essential [8]. Denmark’s experience demonstrates that banning growth promoters, coupled with strict veterinary prescriptions and regular farm audits, can lead to significant reductions in antimicrobial use [21].

Farm-level interventions should prioritize preventive health measures to reduce the need for antibiotics. These include improved hygiene and biosecurity, vaccination programs, optimized nutrition, and selective breeding for disease-resistant livestock. Studies indicate that farms implementing these strategies consistently report lower antibiotic consumption and a reduced prevalence of multidrug-resistant (MDR) bacteria [35,41]. Veterinary education and farmer awareness campaigns also play a critical role. Many farmers, especially in LMICs, are unaware of the long-term consequences of antibiotic misuse or lack access to clear, evidence-based guidelines. Tailored training for both farmers and veterinarians, combined with incentives for responsible antibiotic use, can significantly improve antimicrobial stewardship [35,41]. Finally, promoting alternatives to antibiotics, such as probiotics, bacteriophages, phytogenics, and immunostimulants, offers promising complementary strategies to enhance cattle health and productivity without contributing to resistance development [8,38]. These interventions, supported by clear regulatory frameworks, effective monitoring, and stakeholder engagement, form the foundation of sustainable AMR mitigation in cattle farming. 

Globally, the response to MDR has been inconsistent, with high-income countries like Denmark demonstrating the potential impact of stringent regulation and oversight. By banning antibiotics for growth promotion and investing in comprehensive surveillance, Denmark has achieved measurable reductions in resistance rates [21]. These efforts demonstrate the effectiveness of coordinated, evidence-based policy interventions. In contrast, LMICs face challenges such as weak regulations, economic pressures, and limited veterinary services. Addressing these disparities requires more than simply adopting strategies from high-income countries; it demands context-specific approaches that balance public health priorities with the economic realities of smallholder farmers [27,33].

## 3. Discussion

### 3.1. A Call for Holistic Interventions

Addressing MDR in livestock requires a multifaceted approach grounded in the One Health framework, which integrates human, animal, and environmental health strategies. Enhanced surveillance programs are critical for monitoring resistance trends and identifying high-risk clones, such as *E. coli* ST131, which has been implicated in both livestock and human infections [22]. Regulatory frameworks must enforce stricter controls on AMU while promoting alternatives such as vaccinations, probiotics, and improved husbandry practices to reduce reliance on antibiotics [8,43].

Education and awareness campaigns targeting both farmers and veterinarians are essential for fostering responsible antimicrobial use. Studies have shown that farmers often lack a comprehensive understanding of AMR, emphasizing the need for tailored training programs to address knowledge gaps [35,41]. Advanced molecular tools, such as WGS and metagenomics, can further unravel transmission dynamics and inform evidence-based policy development [21,39]. Promoting sustainable livestock production systems that prioritize animal health and welfare, coupled with robust biosecurity measures, is critical for curbing the spread of MDR pathogens.

AMR in livestock farming presents a significant challenge to global health, with mounting evidence indicating the bidirectional transmission of MDR bacteria among animals, humans, and the environment. The emergence of MDR *E. coli*, *Staph. aureus*, *Salmonella* spp., and other zoonotic pathogens underscores the urgent need for coordinated efforts to mitigate the spread of resistance [32,40]. Likewise, the public health implications of MDR *K. pneumoniae*, CoNS, and *A. baumannii* are significant due to their zoonotic transmission potential, as evidenced by documented human colonization or infections genetically traced back to farm animals [45,46,47]. Such instances highlight the risk posed by livestock-associated reservoirs, particularly for immunocompromised individuals or healthcare environments. Enhanced surveillance and improved diagnostic awareness are essential for early detection and prevention of potential outbreaks originating from agricultural settings. The intensification of livestock production, increasing global demand for animal protein, and widespread antimicrobial misuse have contributed to the proliferation of AMR, exacerbating its economic, clinical, and societal impacts. Therefore, mitigating the spread of these pathogens requires targeted interventions in livestock farming, particularly strengthening farm-level biosecurity, enforcing stringent hygiene protocols, regular cleaning and disinfection of milking equipment, and effective manure management. Implementing nationwide surveillance to detect these pathogens early, coupled with prudent antibiotic stewardship programs limiting critically important antibiotics in animal agriculture, is paramount. Education of farmers on the zoonotic risks associated with these specific MDR pathogens also constitutes a critical component of One Health strategies.

### 3.2. Livestock as Reservoirs for MDR Pathogens

Multidrug-resistant *K. pneumoniae*, CoNS, and *A. baumannii* are increasingly detected in livestock and agricultural environments worldwide. Surveillance efforts have identified carbapenem-resistant *K. pneumoniae* carrying resistance genes such as bla_NDM, bla_OXA-48, or ESBL genes in cattle and poultry farms [48,49]. Methicillin-resistant CoNS (MR-CoNS) are also widespread in dairy and swine operations, highlighting agricultural settings’ significant role in the spread of AMR [50]. A 2023 study conducted in Italy found CoNS in 72.7% of sampled swine and poultry farms (primarily through housefly vectors), with over half of the isolates classified as MDR [50]. CoNS are also common agents of bovine mastitis and frequently colonize farm environments such as milking equipment and bedding, showing high AMR rates. A study from Spain reported that 72.5% of healthy pigs and 60% of pig farmers carried CoNS in their nasal cavities, with 92% of these isolates being MDR [30]. Similarly, in Ethiopian poultry farms, *A. baumannii* was recovered from approximately 15% of chicken litter samples, often exhibiting MDR [51]. These findings emphasize that MDR pathogens are not limited to hospitals but also inhabit food-producing animals, farm soil, water, and even insect vectors, creating potential reservoirs for zoonotic transmission.

Recent genomic and epidemiological studies have traced the movement of MDR pathogens between animals and humans, providing substantial evidence of interspecies transmission. One Health investigations frequently detect overlapping strains or resistance genes, suggesting shared reservoirs. For instance, a 2023 survey of pig farms in Spain identified identical CoNS strains (including rare linezolid-resistant variants) in pigs and farmers, suggesting probable bidirectional transmission on farms [30]. These livestock-associated CoNS carried critical resistance genes, including mecA and cfr (conferring linezolid resistance), highlighting farm environments as reservoirs for clinically significant resistance genes that could be transferred to humans [30]. A genomic survey on a Caribbean island identified a shared ST23 clone of *K. pneumoniae* in both a vervet monkey and a human UTI patient, illustrating how hypervirulent clones can occasionally cross species barriers [52]. An investigation in Switzerland demonstrated a high prevalence of MR-CoNS in livestock (48%) and farmers (49%). Livestock-associated species (e.g., *S. sciuri*, *S. fleurettii*) predominated in animal samples, while human-associated species (*S. epidermidis*, *S. haemolyticus*) were more common among farmers. Researchers cautioned that CoNS from food animals might serve as gene reservoirs, potentially transferring mecA to human pathogens such as *S. aureus* [53]. Additionally, *K. pneumoniae* isolates from dogs in Korea harbored identical CTX-M β-lactamase genes on plasmids as those from human strains, suggesting exchanges between companion animals and humans. In one striking household case, identical MDR *K. pneumoniae* strains carrying the ESBL gene bla_CTX-M-15 were isolated from a healthy dog and its owner, strongly indicating direct zoonotic transmission through close contact [54]. Although companion animals differ from livestock, this scenario underscores zoonotic potential in household contexts. Another noteworthy case occurred on a primate research farm, where a small outbreak of hypervirulent *K. pneumoniae* (capsular serotype K2) emerged among vervet monkeys, causing significant morbidity. While humans were not infected in this event, the monkeys’ reservoir status raised concerns regarding potential spillover to humans [52].

Although overt human outbreaks traced explicitly to livestock CoNS are uncommon, sporadic cases highlight potential animal-to-human transmissions. For example, *S. sciuri*, a CoNS species prevalent among farm animals, has been isolated from human skin infections [53]. Similarly, *A. baumannii* has occasionally caused community-acquired infections in individuals exposed to agricultural environments, as seen in severe pneumonia cases linked to environmental sources like dust inhalation or contaminated water [55,56]. The relative scarcity of large documented outbreaks might partially result from under-detection, as routine diagnostic methods typically do not investigate animal origins for Klebsiella or CoNS infections. Nonetheless, existing case studies and genomic evidence clearly demonstrate a latent zoonotic risk under conducive conditions such as close contact, inadequate hygiene, or raw consumption of animal products.

### 3.3. MDR Transmission and Public Health Implications

Multiple studies have demonstrated the zoonotic potential of AMR pathogens, particularly in dairy farming, where STEC and livestock-associated *Staph. aureus* (LA-MRSA) have been identified in farmers, farm workers, and their surrounding environments [32,41]. The presence of MDR strains, such as CTX-M-15-type ESBL-producing *E. coli* and LA-MRSA ST398, has been linked to severe human infections, including hemorrhagic colitis, HUS, and complicated skin and soft tissue infections [39,40].

A particularly concerning development is the increased resistance to last-resort antibiotics, including colistin and carbapenems, which poses a serious challenge to clinical management [17]. Studies have documented MDR *SH* strains circulating between dairy calves and humans, highlighting the high potential for zoonotic spillover events [21]. WGS analyses have revealed striking genetic similarities between bacterial isolates from livestock and human infections, supporting the hypothesis of inter-host transmission [32]. Furthermore, HGT between commensal and pathogenic bacteria in the human gut microbiome facilitates the dissemination of resistance genes, increasing the risk of community-wide AMR outbreaks [22].

### 3.4. Economic and Clinical Consequences of AMR in Livestock

The economic burden of AMR extends beyond increased healthcare costs and treatment failures to include disruptions in agricultural productivity and international trade. For instance, zoonotic outbreaks caused by MDR pathogens, such as avian influenza and MDR *Salmonella*, have led to billions of USD in losses due to culling, trade restrictions, and decreased consumer confidence in livestock-derived products [21]. Projections suggest that AMR could result in a 3% to 8% annual reduction in livestock production by 2050, disproportionately affecting LMICs where access to veterinary oversight and antimicrobial stewardship programs is often limited [8].

In dairy farming, mastitis caused by MDR *S. aureus* strains significantly impacts milk production, leading to economic losses for farmers and increased reliance on HPCIA for treatment [41]. Studies have shown that many commonly used intramammary antibiotic formulations contain critically important antimicrobial classes, such as cephalosporins and aminoglycosides, which are crucial for human medicine [35]. The overuse of these antibiotics not only accelerates resistance development but also increases the risk of antimicrobial residues in dairy products, further complicating public health efforts to mitigate AMR [8].

Despite growing awareness of AMR risks, many farmers and veterinarians continue to perceive AMU as an essential component of animal husbandry, often prioritizing economic factors and production efficiency over responsible AMU practices [35,41]. Surveys conducted among dairy farmers in Scotland and Switzerland have identified key demographic and behavioral predictors of AMU, with younger farmers and those managing larger herds more likely to engage in high AMU [35]. Additionally, the tendency of farmers to self-prescribe antibiotics or rely on peer recommendations rather than veterinary guidance further complicates AMR mitigation efforts [41].

Veterinarians, as key decision-makers in AMU, also face significant challenges in promoting antimicrobial stewardship. Studies in Denmark have shown that veterinarians often feel pressured to prescribe antimicrobials based on farmers’ preferences rather than scientific evidence, contributing to unnecessary and prolonged antimicrobial courses [35]. Moreover, the introduction of restrictive policies, such as prescription-only antimicrobial sales, has led to resistance within the farming community, with some farmers viewing such regulations as an infringement on their autonomy [41]. The lack of field-generated research tailored to specific livestock settings further hinders veterinarians’ ability to make informed AMU decisions, underscoring the need for locally relevant guidelines and continued professional education [8].

### 3.5. A One Health Approach to AMR Mitigation

Efforts to regulate AMU in livestock have shown varying degrees of success, with high-income countries implementing stricter policies and surveillance systems, while LMICs continue to face significant enforcement challenges [8]. In Denmark, for example, stringent regulations on antibiotic prescriptions have led to measurable reductions in veterinary AMU, yet these policies have also introduced unintended consequences, including hesitancy among veterinarians to prescribe antimicrobials even when clinically justified [35]. Conversely, in LMICs, weak regulatory frameworks and limited veterinary oversight have allowed for the unchecked sale and use of antimicrobials, exacerbating resistance development [8].

The intensification of livestock production in LMICs presents additional hurdles for AMR control. While high-income countries have implemented biosecurity measures and alternative husbandry practices to reduce AMU, these approaches have not been effectively transferred to LMICs, where economic constraints and lack of access to veterinary services remain critical barriers [8]. Addressing these disparities requires a global commitment to improving AMR surveillance, enhancing veterinary education, and fostering interdisciplinary collaboration among stakeholders in the agricultural and health sectors [41].

Given the complexity of AMR transmission at the human–animal–environment interface, a One Health approach is essential to developing sustainable solutions. Strengthening biosecurity measures on farms, promoting judicious AMU practices, and investing in genomic epidemiology tools such as WGS and multilocus sequence typing (MLST) can facilitate early detection and containment of MDR pathogens [32]. Additionally, behavioral interventions targeting farmers and veterinarians, including educational initiatives and incentive-based stewardship programs, have the potential to shift attitudes toward responsible AMU [35].

Collaboration between policymakers, researchers, and industry stakeholders is critical to bridging existing knowledge gaps and implementing evidence-based strategies for AMR mitigation. Future research should focus on developing region-specific AMU guidelines, improving access to veterinary care in LMICs, and enhancing public awareness of the risks associated with antimicrobial misuse. By adopting a One Health framework, it is possible to balance the need for effective disease management in livestock with the long-term goal of preserving antimicrobial efficacy for future generations.

## 4. Conclusions

The emergence and persistence of MDR bacteria in livestock farming pose significant public health, economic, and ecological challenges. MDR pathogens originating from cattle not only limit treatment options in human medicine—leading to increased morbidity and mortality—but also threaten economic stability. Antimicrobial resistance is projected to reduce livestock production by 3% to 8% annually by 2050, disrupting global food systems and placing severe financial burdens on farmers. The growing prevalence of zoonotic outbreaks linked to MDR bacteria further escalates healthcare costs, increases hospitalizations, and reduces the efficacy of last-resort antibiotics such as colistin, underscoring the urgent need for action. This issue reflects broader systemic failures, including the over-reliance on antibiotics as quick fixes, the undervaluing of preventive measures such as vaccination and biosecurity, and the fragmented accountability across the agricultural, healthcare, and regulatory sectors.

Addressing MDR in livestock requires a holistic, long-term strategy that prioritizes sustainability over short-term gains. The interconnectedness of human, animal, and environmental health highlights the necessity of an integrated One Health approach. Targeted interventions are crucial to tackling this crisis, including robust surveillance programs to track resistance trends, stricter regulations on antimicrobial use, and the promotion of hygienic farming practices to curb resistance development. Additionally, fostering collaboration among farmers, veterinarians, policymakers, and researchers is essential to reducing transmission risks and establishing sustainable livestock production systems. Ultimately, the cost of inaction far exceeds the investment needed for change. MDR in livestock farming is not just a scientific or economic challenge—it is a moral imperative that requires global cooperation and innovative solutions.

Finally, addressing AMR in agriculture is crucial for safeguarding public health and ensuring sustainable livestock production. However, tackling this complex crisis requires a holistic approach that transcends traditional disciplinary boundaries. The One Health framework, endorsed by organizations such as the WHO and FAO, highlights the interdependence of human, animal, and environmental health in combating AMR. One Health initiatives advocate for improved antibiotic stewardship in agriculture and livestock farming, as well as the promotion of alternatives such as vaccines and probiotics to reduce reliance on antimicrobials [38] and the implementation of stringent regulatory measures. Despite progress, significant disparities in global regulations and enforcement highlight the need for coordinated efforts to curb antibiotic misuse and protect the efficacy of these vital drugs.

## Figures and Tables

**Table 1:** Overview of antimicrobials shared between human and veterinary medicine [7].

Category	Organism/Source	AMR Genes	Comments	WHO Ranking	OIE Ranking	Animal Species
Carbapenemase Genes in Gram-negative Bacteria	*Escherichia coli* (cows)	bla_KPC, bla_NDM, bla_OXA-48, bla_VIM, bla_IMP	Found in *E. coli* isolated from cattle; transferable via plasmids.	CI	VCIA	Cows
Carbapenemase Genes in Gram-negative Bacteria	*Klebsiella pneumoniae* (cows)	bla_NDM, bla_OXA-48	Often associated with multidrug resistance (MDR).	CI	VCIA	Cows
Carbapenemase Genes in Gram-negative Bacteria	*Pseudomonas aeruginosa* (cows)	bla_VIM, bla_IMP	Notably found in environmental or hospital-related isolates from animal settings.	CI	VCIA	Cows
AMR Genes in Staphylococci from Animal and/or Human Origin	*Staphylococcus aureus* (human)	mecA, mecC, blaZ, erm(A), erm(C), tet(M), dfrG, aac(6′)-Ie-aph(2″)-Ia	Common genes conferring resistance to beta-lactams, macrolides, tetracyclines, and aminoglycosides.	CI	VHIA	Humans
AMR Genes in Staphylococci from Animal and/or Human Origin	*Staphylococcus aureus* (animal)	mecC, erm(C), tet(K), fusB	Resistance to beta-lactams, macrolides, and tetracyclines observed in livestock isolates.	HI	VHIA	Cows, Pigs
AMR Genes in Staphylococci from Animal and/or Human Origin	*Staphylococcus epidermidis*	mecA, mecC, aac(6′)-Ie-aph(2″)-Ia, dfrG	Found in both human and animal isolates; significant role in coagulase-negative staphylococci resistance.	HI	VHIA	Humans, Animals
Common AMR Genes in Clostridium, Enterococci, and Staphylococci	*Clostridium difficile*	tet(M), erm(B)	Resistance to tetracyclines and macrolides; occasionally linked to transposons.	CI	VCIA	Multiple
Common AMR Genes in Clostridium, Enterococci, and Staphylococci	*Enterococcus faecalis*	vanA, vanB, aac(6′)-Ie-aph(2″)-Ia, erm(B), tet(M)	Resistance to glycopeptides, aminoglycosides, macrolides, and tetracyclines.	CI	VCIA	Humans, Livestock
Common AMR Genes in Clostridium, Enterococci, and Staphylococci	*Enterococcus faecium*	vanA, erm(B), tet(M), aac(6′)-Ie-aph(2″)-Ia	Vancomycin-resistant genes prevalent in hospital and animal settings.	CI	VCIA	Humans, Animals
Common AMR Genes in Clostridium, Enterococci, and Staphylococci	*Staphylococcus aureus*	mecA, mecC, blaZ, erm(C), tet(M)	Common resistance genes in both human and animal isolates.	CI	VHIA	Humans, Livestock

WHO Ranking: CI = Critically Important, HI = Highly Important. OIE Ranking: VCIA = Veterinary Critically Important Antimicrobial, VHIA = Veterinary Highly Important Antimicrobial. Animal Species: Indicates the animals in which the antibiotic is applied.

## Data Availability

No new data were created or analyzed in this study.
